# Securing gait recognition with homomorphic encryption

**DOI:** 10.1038/s41598-025-14047-3

**Published:** 2025-08-12

**Authors:** Marina Banov, Domagoj Pinčić, Diego Sušanj, Kristijan Lenac

**Affiliations:** 1 Faculty of Engineering, University of Rijeka, Vukovarska 58, Rijeka, 51000 Croatia; 2 Center for Artificial Intelligence and Cybersecurity, University of Rijeka, Radmile Matejčić 2, Rijeka, 51000 Croatia; 3 Faculty of Engineering, Juraj Dobrila University of Pula, Alda Negrija 6, Pula, 52100 Croatia

**Keywords:** Secure biometrics, Gait recognition, Homomorphic encryption, Privacy preserving computation, Deep learning, Engineering, Mathematics and computing, Computer science, Information technology, Software

## Abstract

Biometric identification systems offer strong security by relying on unique personal traits. At the same time, they raise significant privacy concerns because compromised biometric data cannot be revoked. This paper explores the use of homomorphic encryption (HE) as a means to protect biometric data during classification and reduce the risk of exposing sensitive information. Our system comprises a feature extractor which operates locally and a classifier which processes encrypted data. We demonstrate the feasibility of our approach on a gait recognition task, employing a vision transformer as a feature extractor and training several HE-compatible classifiers. Through a comprehensive statistical analysis, we evaluate the impact of HE on accuracy and computational complexity, especially with different activation functions and their polynomial approximations. Our results demonstrate the feasibility of secure and accurate gait recognition using HE, while highlighting the trade-off between security and performance.

## Introduction

Biometric identification systems provide a secure way to distinguish individuals based on their physical or behavioral traits, such as fingerprint, face, or gait features. Biometric traits are notably difficult to replicate or forge, which makes them a secure form of identification. Their use could also reduce the need for remembering complex passwords or carrying physical tokens and results in a more seamless user experience. These qualities make biometric systems highly reliable and convenient for secure identification, and can even complement traditional systems in multi-layered approaches. However, the same properties that make biometric data valuable also make it more dangerous if breached, as it directly links to a person’s identity and cannot be changed like a password. Once an individual’s biometric data has been exposed, they remain permanently vulnerable to identity theft and unauthorized access. The nature of the associated privacy risks can vary across different biometric modalities, as some modalities are more suitable for certain applications than others.

Gait recognition, in particular, has attracted interest due to its unobtrusive, distance-based nature. It does not require subject cooperation, it is resistant to imitation and is, therefore, well-suited for applications like surveillance, forensic identification, and remote authentication^1,2^. These use cases have motivated the development of increasingly accurate recognition systems, often driven by deep learning models. However, the resulting feature representations may unintentionally encode sensitive attributes beyond the intended identification task, including race, gender, age, and health conditions^3^. This calls for privacy-preserving techniques that can balance functionality with protection.

According to Meden et al.^4^, there are two ways of addressing privacy concerns related to biometric data: focusing on encryption schemes or minimizing unauthorized data access, and modifying biometric data to limit the exposure of intrinsic information. The authors emphasize that, unless biometric matching is performed on encrypted data, sensitive information can be exposed once it is decrypted for processing, and then focus on the latter approach. In this manuscript, we explore homomorphic encryption﻿ (HE) as a powerful solution that allows biometric matching to be performed directly on encrypted data, reducing privacy and security risks.

A HE scheme is an asymmetric cryptosystem with an additional efficient evaluation algorithm that operates on a set of ciphertexts and produces an encrypted result. When decrypted, this result matches the result of the operations as if they had been performed on plaintexts. This property was first observed with the RSA encryption scheme^5^, which supported multiplication on encrypted data. Later developments, including other partially homomorphic encryption (PHE) schemes and somewhat homomorphic encryption (SHE), marked significant steps toward practical HE, but with their own limitations. Finally, in 2009, Gentry introduced the concept of bootstrapping and presented the first fully homomorphic encryption (FHE) scheme^6^, which theoretically allows for arbitrary secure computations. While Gentry’s original scheme was computationally intensive and not practically feasible, it drew significant attention to the field. Thanks to algorithmic improvements like handling floating-point numbers^7^, HE has since become more suitable for various applications, including machine learning (ML). Additionally, the development of user-friendly software frameworks^8^ has made it more accessible to a wider range of researchers and developers.

While HE seems like a promising solution for secure biometric identification, it still faces challenges related to efficiency and computational overhead. This paper explores the potential of HE for biometric recognition and addresses the trade-off between security and performance. We build upon prior work that utilized vision transformers (ViTs) for gait recognition^9^ and introduce HE to our pipeline. However, the architecture proposed in our earlier work is not suitable for encrypted computations in its original form. It is based on deep models that would lead to significant computational overhead and it involves non-linear operations which are not supported in the encrypted domain. To address this, we divide our model into two parts: a feature extractor (FE) and a classifier (CLS)^10,11^. While both parts handle sensitive information, the FE is assumed to compute the feature vectors locally within a secure environment, whereas the CLS can be performed remotely or in any location, provided it operates on encrypted data to maintain privacy. We modify the classifier to support HE and conduct a comprehensive statistical analysis to evaluate the impact of HE on both accuracy and computational complexity.

**Fig. 1:**
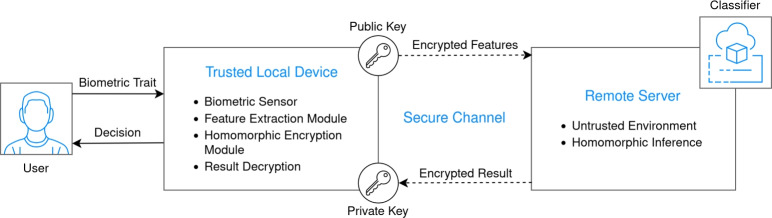
System architecture showing local encryption of biometric features and remote homomorphic classification.

To demonstrate the applicability of our approach, consider a use case where a user wishes to submit biometric data for classification without exposing sensitive information. In this system, we assume that the biometric feature vectors are computed on a trusted local device, such as a certified sensor or a secure enclave, that ensures the features are derived from authentic biometric input. These feature vectors are then encrypted using HE and sent over a secure channel (e.g., TLS) to a privacy-preserving computation node, where the classification process takes place. While the input data is encrypted, the classifier’s parameters remain in plaintext, which is crucial for maintaining computational efficiency^12,13^. This architecture, as illustrated in Fig. 1, allows users to submit their data for classification without compromising their privacy.

In this work, we present the following contributions:

- We introduce an HE-friendly classifier for gait recognition, utilizing the CKKS^7^ scheme.
- We systematically evaluate the trade-offs between computational overhead and accuracy when performing gait recognition in both plaintext and encrypted domains.
- We conduct extensive statistical analyses to assess the impact of different activation functions and their polynomial approximations on classification performance.

Through this work, we focus on the practical integration of existing homomorphic encryption techniques within a gait recognition pipeline. Our central hypothesis is that privacy can be enhanced through HE during classification, provided that securely extracted features are available, and that this can be achieved with minimal accuracy loss, an outcome we empirically confirm in this study.

This paper is structured as follows: “Related work” section provides a review of related work. “Methods” section details the dataset used in our research, the development of our HE-friendly classifier, and our experimental setup. In “Results” section, we address three key research questions regarding the impact of HE on accuracy and computational efficiency, supported by detailed statistical analyses of our results. Finally, “Conclusion” section summarizes our findings and discusses the implications of our work.

## Related work

Since Gentry’s first FHE scheme, research on HE in machine learning has gained considerable attention, with much of the work focusing on theoretical developments and experiments using general-purpose benchmark datasets^14,15^. However, recent studies have started exploring practical applications of HE in various domains. This trend is evident in fields such as genomics and bioinformatics^16–18^, as well as industries such as agri-food, where a study by Onoufriou et al.^19^ showcased the potential of deploying HE in convolutional neural networks (CNNs). Jia et al.^20^ presented a successful attempt to improve the efficiency of privacy-preserving image classification through selective encryption of only feature-rich chunks of data, specifically conducted on images of steel surface defects. Although the specific approaches used in these works may not be directly applicable to biometric data, their shared efforts in adapting ML models, particularly CNNs, for homomorphic computations provide guidelines which are relevant to our study.

In the field of biometrics, most existing works that use HE for matching and identification focus on modalities such as facial and iris recognition. Here, we outline some relevant studies. For a more in-depth discussion, readers are referred to a comprehensive review on privacy-preserving biometrics using HE^21^.

Drozdowski et al.^22^ investigated the application of HE to face identification systems, providing a detailed analysis of its feasibility and effectiveness in performing biometric matching. Pradel and Mitchell^23^ expanded on this by developing a privacy-preserving biometric matching framework using HE, effectively balancing computational efficiency with strong privacy protection. Yang et al.^24^ proposed a face recognition system incorporating HE to ensure data confidentiality while maintaining high recognition accuracy, even under challenging conditions such as various degrees of face bias. Engelsma et al.^25^ introduced HERS, a framework leveraging HE for secure representation-based searches, enabling privacy-preserving facial recognition at scale. Building on these efforts, Nocker et al.^11^ introduced HE-MAN, an open-source framework for privacy-preserving ML inference with HE and ONNX models, demonstrating its effectiveness on the facial identification task.

Furthermore, a number of works have investigated the use of HE to protect the confidentiality of iris templates. Morampudi et al.^26^ proposed a privacy-preserving iris authentication method using FHE, which encrypts iris templates to ensure confidentiality while enabling secure biometric matching. Kumar et al.^27^ introduced BMIAE, a blockchain-based multi-instance iris authentication system that uses additive ElGamal HE to ensure the confidentiality of iris templates.

Expanding on single-modal approaches, multi-modal biometric systems that fuse multiple biometric traits have also adopted HE techniques. Gomez-Barrero et al.^28^ proposed a general framework for multi-modal biometric template protection using HE, ensuring that only encrypted data is handled during verification. Sperling et al.^29^ introduced HEFT, a secure method for fusing and matching encrypted biometric templates using FHE, and evaluated its performance on both face and voice biometrics. Singh et al.^30^ investigated the use of FHE in multimodal iris and face recognition systems, where iris and face features are first fused and then encrypted, ensuring secure matching of the combined encrypted templates. Vallabhadas et al.^31^ proposed a multi-modal biometric authentication system that uses FHE to secure iris and fingerprint templates, incorporating a rotational invariant technique to further enhance recognition accuracy.

The application of HE to gait recognition has received comparatively less attention, though some recent efforts have started to address this gap. For example, Lin et al.^32^ presented a privacy-preserving scheme for running deep neural networks (DNNs) on encrypted data, even during the training phase. However, this work is theoretical in nature and lacks experimental results. A notable study by Castro et al.^33^ combined PHE and cancelable biometrics with a long short-term memory (LSTM) neural network to protect gait data in the context of early dementia diagnosis. Although focused on medical diagnosis, this work addresses the challenge of balancing privacy and performance.

Beyond HE, several works have explored alternative specialized mechanisms for increasing privacy when working with gait data. Naz et al.^34^ applied hardware-based optical encryption at the data capture stage to prevent identifiable imagery from being digitized, though only for binary abnormal gait detection. Delgado-Santos et al.^35^ used unsupervised learning to disentangle identity features from sensitive attributes, adapting techniques from style transfer to generate privacy-preserving gait representations. Parashar and Shekhawat^36^ focused on reversible anonymization of gait silhouettes for dataset protection, employing image processing techniques rather than traditional template protection methods. Su et al.^37^ proposed stochastic orthogonal transformations for protecting biometric templates in gait recognition systems, coupled with a biometric-based encryption scheme for secure communication. These approaches mostly rely on obfuscation, transformation, or anonymization and lack privacy guarantees provided by established cryptographic methods.

Formal privacy-preserving techniques have been studied in other biometric modalities, offering promising design patterns for secure data processing. Although their direct integration into gait recognition is still emerging, we briefly outline these methods, discuss their respective advantages and typical use cases, and present any gait-related work. For a broader discussion of their application across biometric systems, readers are referred to a more comprehensive review^38^.

Bloom filters are a lightweight cryptographic technique often used in biometrics to check if a user’s data matches some stored template. These hash-based data structures are efficient and compact but can lead to collisions and false positives. Bloom filters have been successfully applied in gait recognition for data obtained by wearable sensors^39^. Template protection schemes like cancelable biometrics and fuzzy commitments secure stored biometric data in cases where biometric templates may be compromised. They are particularly effective in authentication tasks, as they provide revocability, unlinkability, and error-tolerant matching. Security of fuzzy commitment schemes has previously been evaluated in gait-based systems^40^. Secure multi-party computation (SMPC) and secret sharing are suitable in collaborative scenarios where several participants want to process joint biometric data without disclosing sensitive information. SMPC has been implemented in live biometric systems^41^ but, to the best of our knowledge, not with gait data. However, there have been efforts using federated learning to address privacy concerns during distributed training^42^, which should ideally be combined with other cryptographic measures to offer stronger guarantees in mitigating membership inference attacks. Differential privacy (DP) ensures that the inclusion of an individual’s data does not significantly influence the results of large datasets analyses. Though not for identification, DP has been used in human activity recognition to protect sensitive information derivable from seemingly anonymous sequential movement data^43^, suggesting its potential for gait.

Compared to these approaches, homomorphic encryption presents a compelling solution for our gait recognition system. HE offers higher security than Bloom filters and allows arbitrary computations on encrypted data. This is a significant advantage over template protection methods that primarily secure stored data and have limited computational capabilities. While SMPC is powerful in multi-party scenarios, our framework does not involve collaborative data processing. Similarly, differential privacy is effective in protecting large-scale statistical analyses but its inherent noise introduction makes it unsuitable for the precise individual inferences our system requires. This motivates our work to explore the feasibility of integrating HE into gait recognition systems through empirical evaluation.

## Methods

In this manuscript, we conducted experiments on a widely used benchmark dataset in the field of gait recognition, CASIA-B^44^. The dataset includes 124 subjects, captured under three different walking conditions and from 11 different viewing angles. Walking conditions encompass normal walking (NM) with six sequences per subject, walking with a bag (BG) with two sequences per subject, and walking with a coat or a jacket (CL) also with two sequences per subject. On average, each subject contributes a total of 110 sequences. In our case, each sequence corresponds to a single GEI image^45^ representing an individual’s gait cycle, resulting in nearly 13,600 images collectively. In our experiments, after the FE is trained on the data from the first 74 subjects, the gait features can be extracted for the remaining 50 subjects and used for classification. Additionally, within each subject’s data, we divided the sequences into two sets: gallery and query. The gallery set comprises the first 4 sequences of the NM modality for training. The query set consists of the remaining 2 NM sequences, along with the 2 sequences of BG and CL modalities for evaluation.

We employ the self-supervised model DINO as a FE to capture gait features from unlabeled training data^9^. DINO utilizes the ViT architecture as its backbone, offering notable advantages over traditional CNN-based models^46^. The combination of a self-supervised learning approach with ViTs enables the model to effectively differentiate target objects from backgrounds without explicit supervision, which is particularly advantageous for gait recognition as it enhances the model’s ability to focus on subtle motion patterns while ignoring irrelevant noise. DINO operates using a self-distillation strategy in a student-teacher framework, where both networks share the same architecture but have different parameter update rules. The model is trained to produce consistent feature representations across different augmented views of the same input, thereby encouraging invariance to transformations. DINO promotes clustering of semantically similar instances in the feature space without requiring identity labels, effectively drawing feature vectors of similar patterns closer in the embedding space based on their visual and structural consistency. The inputs to the DINO model are GEI images, which are processed to generate fixed-dimensional feature vectors representing the subject’s gait.

**Fig. 2:**
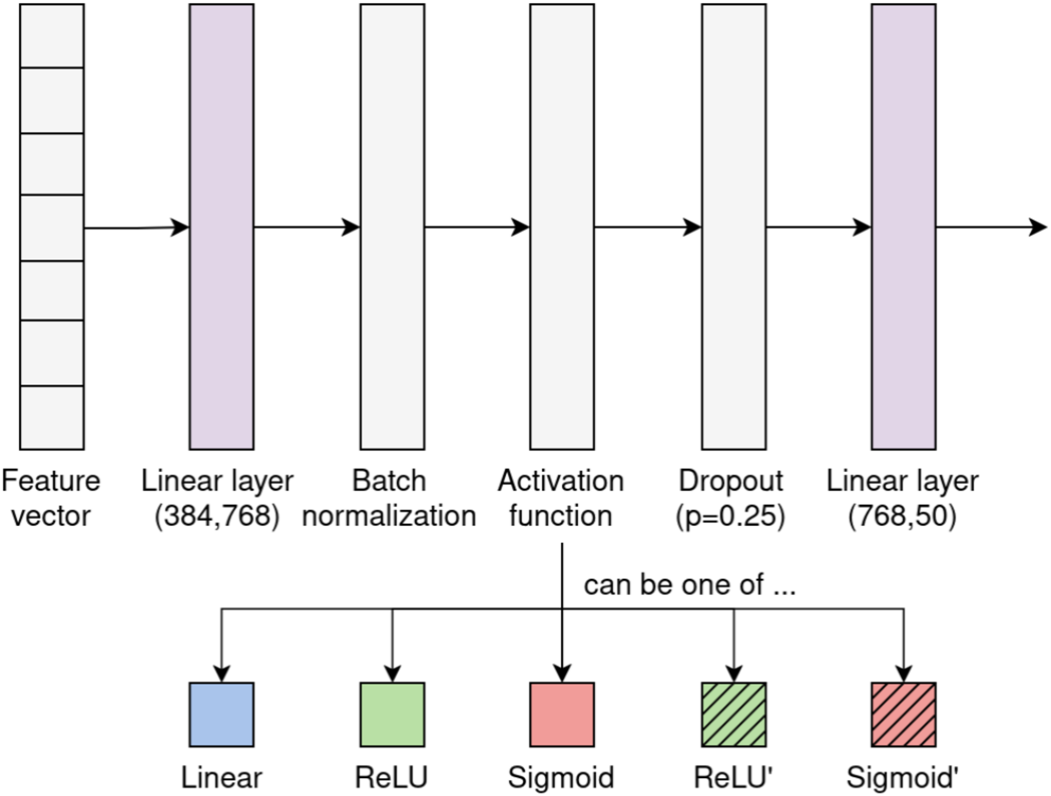
Shared architecture of the five classifiers with different activation functions.

Next, a simple fully connected neural network (FCNN) classifier is trained on gallery samples and evaluated on query samples using the features extracted by the DINO FE. The classifier’s architecture is inspired by our prior research and employs the same hyperparameters as detailed in the original work^9^. As shown in Fig. 2, it consists of two linear layers, batch normalization, an activation function, and dropout. The classifier outputs a probability distribution over all known subject identities, with the identity corresponding to the highest probability selected as the final prediction. We employed five variants of this classifier, each designed to evaluate different activation functions and their compatibility with the HE framework:

- $\text {CLS}_\text {Linear}$: This classifier uses the linear activation function $f_\text {Linear}(x)=x$, which does not change the scores but relies on the loss function to induce the correct behavior. This variant serves as a control mechanism to establish a baseline for comparison.
- $\text {CLS}_\text {ReLU}$: This classifier uses the ReLU activation function $f_\text {ReLU}(x)=max(0,x)$. Due to its non-linearity, it cannot be directly used with HE and requires a polynomial approximation.
- $\text {CLS}_\text {Sigmoid}$: This classifier uses the sigmoid activation function $f_\text {Sigmoid}(x)=\frac{1}{1+e^{-x}}$. Like ReLU, it cannot be directly used with HE and requires a polynomial approximation.
- $\text {CLS}_\text {ReLU'}$: This classifier uses the function $f_\text {ReLU'}(x)=x^2$, a low-degree polynomial commonly used in homomorphic encryption contexts as a computationally efficient alternative for ReLU. While $x^2$ is not a strict approximation of ReLU, it preserves some aspects of the non-linearity and is computable under HE^47^. For brevity, we refer to it as a polynomial approximation of ReLU.
- $\text {CLS}_\text {Sigmoid'}$: This classifier uses the function $f_\text {Sigmoid'}(x)=-0.004\cdot x^3 + 0.197\cdot x + 0.05$, a low-degree polynomial approximation of the sigmoid activation function, chosen to ensure compatibility with the HE framework^48^. Although such approximations may yield inferior results in more complex networks and tasks^49^, they provide sufficiently accurate results while reducing computational complexity.

**Fig. 3:**
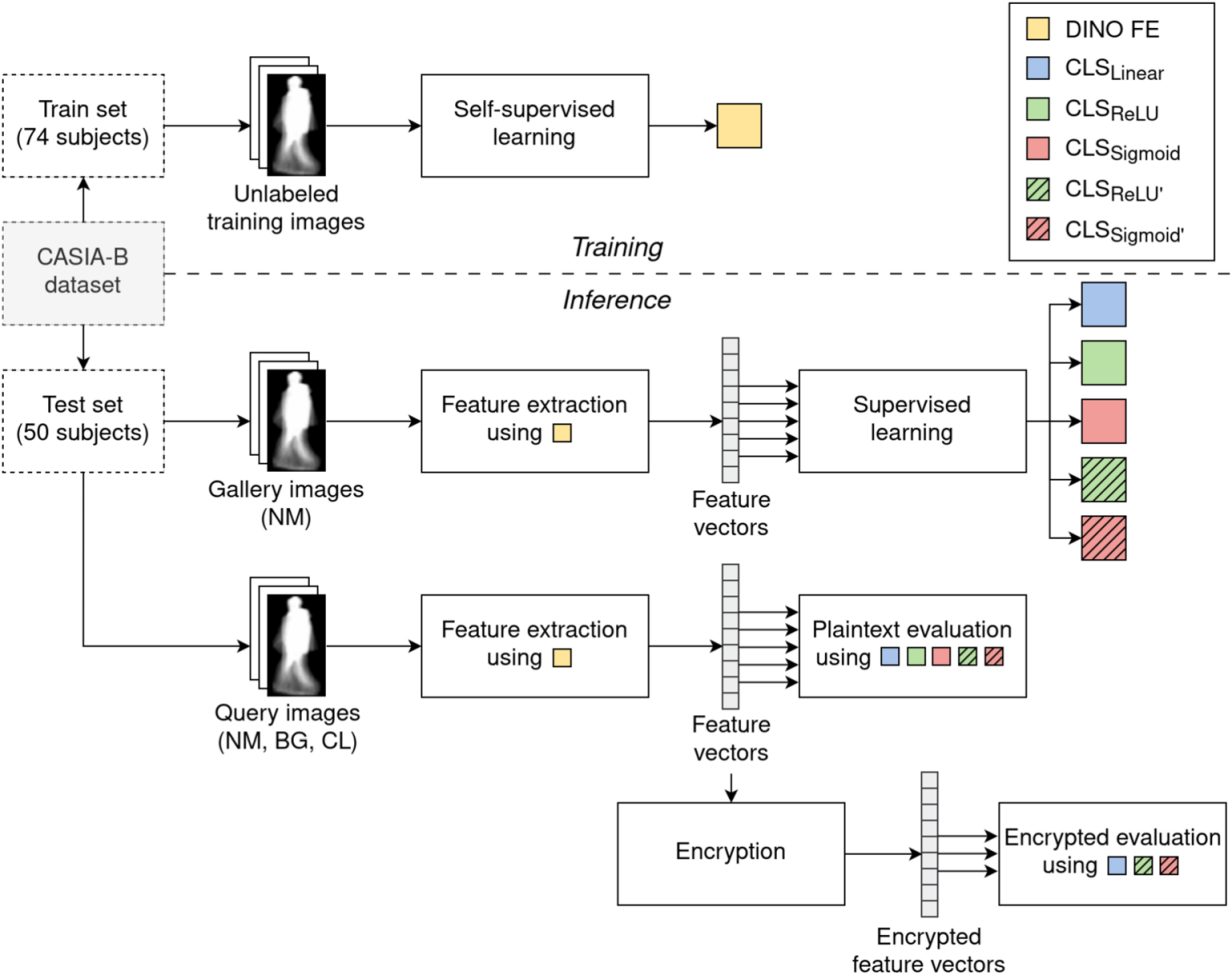
Our processing pipeline, including training and inference in the plaintext and encrypted domain.

All classifiers were trained for 100 epochs, with a batch size of 128. We used the cross-entropy loss function and the Adam optimizer with a learning rate of 0.0005. A common approach is to train the model with plaintext data and use the unencrypted weights on the encrypted data points during evaluation^12,13,17–19^. In fact, after training, we can evaluate the query set in both plaintext and encrypted domains, which allows us to compare whether the classification accuracy is maintained with the added benefit of preserving privacy through HE. A visualization of our processing pipeline can be found in Fig. 3.

During the encrypted evaluation, we deployed the CKKS scheme^7^ provided by TenSEAL^8^, which relies on the Microsoft SEAL framework. The CKKS scheme supports arithmetic operations on real-value data and can be applied to tensors for ML tasks. It is important to carefully select the scheme’s parameters since they directly influence the security, efficiency, and accuracy of the computations performed in the encrypted domain. During the parameter selection process, we ensured that our scheme met a $\lambda =128$ bit security level, according to standard guidelines and our preliminary research findings^11,50,51^. We chose a polynomial modulus degree $N=8192$ and a coefficient modulus chain with bit sizes:

1 $$\begin{aligned} \bigl (q_i\bigr )^{d_m+1}_{i=0}=\bigl (36,\ \underbrace{ 28,\ 28,\ ...,\ 28 }_{ d_m~\text {times}},\ 36\bigr ). \end{aligned}$$

The multiplicative depth $d_m$ is derived from the classifier and varies according to the employed activation function: $\text {CLS}_\text {Linear}$ had a multiplicative depth $d_m=3$, $\text {CLS}_\text {ReLU'}$ led to $d_m=4$, and $\text {CLS}_\text {Sigmoid'}$ resulted in $d_m=5$. Additionally, we determined the scaling factor $\Delta =2^{28}$.

All five classifiers were trained and evaluated 30 times on the CASIA-B dataset in the plaintext domain, following the predefined large-sample subject-wise split. This approach was chosen over *k*-fold cross-validation to ensure comparability with other works in the field of gait recognition. Due to the randomness introduced by the dropout layer during training, multiple runs were necessary to mitigate variance in performance. Additionally, $\text {CLS}_{\text {Linear}}$, $\text {CLS}_{\text {ReLU'}}$, and $\text {CLS}_{\text {Sigmoid'}}$ were further evaluated 30 times in the encrypted domain. This allowed us to conduct comprehensive statistical analyses and draw reliable conclusions regarding the performance differences between the activation functions and across both domains.

## Results

In this section, we present the summarized data derived from our experiments and outline how it will be analyzed to address three distinct research questions. Table 1 displays the means and standard deviations of the accuracy scores achieved by each classifier in plaintext and encrypted domain. The overall mean accuracy is influenced by the inherent variability within the CASIA-B dataset across different angles and modalities. As detailed in our previous work, the NM and BG modalities are relatively easier to classify, with $\text {CLS}_\text {ReLU}$ achieving mean accuracies of 0.997 and 0.783, respectively. In contrast, the CL modality poses a significant challenge due to its effect on gait patterns, achieving a mean accuracy of 0.276, leading to a lower overall mean accuracy for all classifiers^9^.

**Table 1:** Classifier accuracy scores by evaluation type.

	Plaintext evaluation			Encrypted evaluation		
	N	Mean	SD	N	Mean	SD
$\text {CLS}_{\text {Linear}}$	30	0.610	0.006	30	0.605	0.006
$\text {CLS}_{\text {ReLU}}$	30	0.618	0.005	N/A	N/A	N/A
$\text {CLS}_{\text {Sigmoid}}$	30	0.632	0.004	N/A	N/A	N/A
$\text {CLS}_{\text {ReLU'}}$	30	0.595	0.009	30	0.589	0.009
$\text {CLS}_{\text {Sigmoid'}}$	30	0.634	0.005	30	0.632	0.005

In the upcoming subsections, we investigate the following research questions:

1. *Which activation function yields the highest accuracy?* First, we analyze the data obtained from three base activation functions ($\text {CLS}_{\text {Linear}}$, $\text {CLS}_{\text {ReLU}}$, and $\text {CLS}_{\text {Sigmoid}}$) in plaintext evaluation. This decision was made to assess their direct impact on classifier performance, without the confounding effects introduced by polynomial approximations and HE.
2. *Do polynomial approximations provide comparable accuracy to the original activation functions?* Next, we compare the accuracy of the original activation functions to their respective polynomial approximations. Specifically, we examine the pairs $\text {CLS}_{\text {ReLU}}$ vs. $\text {CLS}_{\text {ReLU'}}$ and $\text {CLS}_{\text {Sigmoid}}$ vs. $\text {CLS}_{\text {Sigmoid'}}$. Again, we use the data solely from plaintext evaluation, since classifiers using the original activation functions could not be directly evaluated in the encrypted domain within our framework.
3. *How does classifier evaluation in the encrypted domain compare to plaintext evaluation?* Finally, we examine whether there is a significant difference in classifier performance between plaintext and encrypted evaluation in terms of accuracy and discuss the computational overhead introduced by HE. Here, we use the data obtained from $\text {CLS}_{\text {Linear}}$, $\text {CLS}_{\text {ReLU'}}$, and $\text {CLS}_{\text {Sigmoid'}}$ in both domains.

### Accuracy comparison across activation functions

A repeated-measures ANOVA was performed to evaluate the effect of three activation functions ($f_\text {Linear}$, $f_\text {ReLU}$, and $f_\text {Sigmoid}$) on the classifier’s accuracy.

Mauchly’s test indicated that the assumption of sphericity had been met, $\chi ^2(2)=0.71, \ p=.70$. The effect of the chosen activation function on the classifier’s accuracy was significant at the $\alpha =.05$ level:

$$\begin{aligned} F(2, 58)=221.38, \ p<.001. \end{aligned}$$

The effect size ($\eta ^2_p=.88$) indicated a large effect.

Post-hoc pairwise comparisons with a Bonferroni adjustment indicated that $\text {CLS}_\text {Sigmoid}$ achieved significantly higher accuracy than both $\text {CLS}_\text {ReLU}$ ($p<.001$) and $\text {CLS}_\text {Linear}$ ($p<.001$), while $\text {CLS}_\text {ReLU}$ scored significantly higher than $\text {CLS}_\text {Linear}$ ($p<.001$).

These findings suggest that $f_\text {Sigmoid}$ leads to highest accuracy, followed by $f_\text {ReLU}$, and lastly, $f_\text {Linear}$. The effect size confirms these differences are practically significant.

### Accuracy comparison for polynomial approximations

A paired samples *t*-test was performed to compare the accuracy achieved by classifiers featuring $f_\text {ReLU}$ and its polynomial approximation $f_\text {ReLU'}$. The results indicated that the accuracy for $\text {CLS}_\text {ReLU}$ was significantly higher than for $\text {CLS}_\text {ReLU'}$:

$$\begin{aligned} t(29)=12.31, \ p<.001. \end{aligned}$$

Similarly, a paired samples *t*-test was performed to compare the accuracy achieved by classifiers featuring $f_\text {Sigmoid}$ and its polynomial approximation $f_\text {Sigmoid'}$. The results indicated that the accuracy for $\text {CLS}_\text {Sigmoid}$ was significantly lower than for $\text {CLS}_\text {Sigmoid'}$:

$$\begin{aligned} t(29)=-10.04, \ p<.001. \end{aligned}$$

### Classifier evaluation in plaintext and encrypted domains

A two-way repeated-measures ANOVA (namely, a $3 \times 2$ RM ANOVA) was performed to evaluate the effects of three activation functions ($f_\text {Linear}$, $f_\text {ReLU'}$, and $f_\text {Sigmoid'}$) and evaluation type (plaintext or encrypted) on the classifier’s accuracy.

Mauchly’s test indicated that the assumption of sphericity had been violated for the activation function factor, ${\chi ^2(2)=13.52,} \ p=.001$. Sphericity could not be assessed for the evaluation type factor as it consisted of only two levels. The assumption of sphericity had been met for the interaction between the two factors, $\chi ^2(2)=5.55, \ p=.06$. Therefore, the degrees of freedom were corrected for factors exhibiting violations of sphericity using Greenhouse-Geisser estimates of sphericity $(\varepsilon =.72)$, while the original degrees of freedom were retained for factors meeting the assumption.

The results indicated a significant main effect for the choice of activation function:

$$\begin{aligned} F(1.45, 41.94)=276.74, \ p<.001, \ \eta ^2_p=.91, \end{aligned}$$

a significant main effect for evaluation type:

$$\begin{aligned} F(1, 29)=194.55, \ p<.001, \ \eta ^2_p=.87, \end{aligned}$$

and a significant interaction between the two factors:

$$\begin{aligned} F(2, 58)=18.35, \ p<.001, \ \eta ^2_p=.39. \end{aligned}$$

Given the significant interaction effect between independent variables, a simple main effects analysis was performed, indicating that the accuracy was significantly lower for encrypted evaluation than for plaintext evaluation in all levels of the activation function factor ($p<.001$ for all comparisons). This suggests that the evaluation type has a consistent and statistically significant impact on the accuracy regardless of the chosen activation function.

Having examined the accuracy of our classifiers under plaintext and encrypted evaluation, we now analyze the required computation time. We conducted our experiments on a machine equipped with an AMD Ryzen 7 5700X CPU, featuring 8 cores and 16 threads, coupled with 32 GB of DDR4 RAM, running an Ubuntu 22.04 LTS operating system.

For plaintext evaluation, the computation time was low and consistent across different activation functions, with the entire query dataset (3298 instances) processed in less than a second on average $(\text {Mean}=0.04, \text {SD}<0.01)$, and a single instance in $1.2\times 10^{-5}$ seconds on average.

Homomorphically encrypted evaluation exhibited significantly longer computation times, even with the implementation of parallelization techniques using 10 threads. Table 2 presents the average HE computation time for the entire dataset as well as for individual instances, giving relevant insight for both batch processing scenarios and real-world applications where individual instances are evaluated separately.

**Table 2:** Computation times for encrypted classification, presented in hh:mm:ss.ss format and abbreviated to mm:ss.ss or ss.ss.

	N	Entire dataset		Per instance	
		Mean	SD	Mean	SD
$\text {CLS}_{\text {Linear}}$	30	01:03:28.73	03:17.19	08.42	00.41
$\text {CLS}_{\text {ReLU'}}$	30	01:18:41.19	00:06.63	10.61	00.06
$\text {CLS}_{\text {Sigmoid'}}$	30	01:32:34.97	03:13.01	12.42	00.51

The observed variations in computation times between classifiers are directly linked to their computational complexity. Given that all classifiers shared identical parameters except for their activation functions, their multiplicative depths determined the overall processing time. This explains the linear increase in computation time which aligned with the increment in multiplicative depth across the classifiers.

**Fig. 4:**
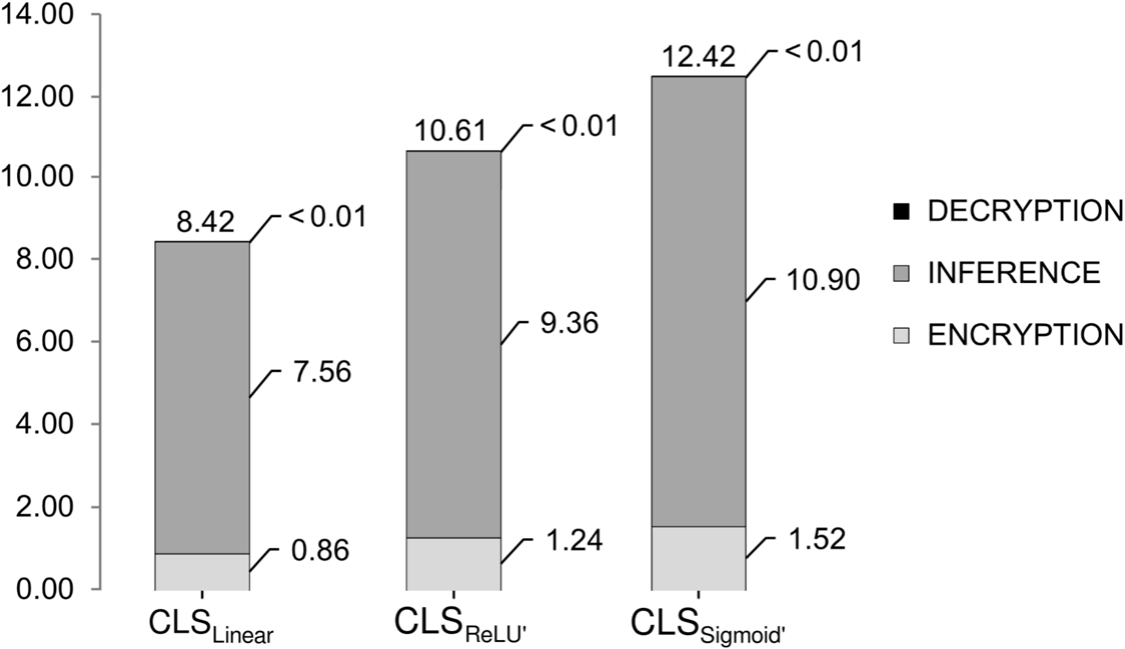
Average processing times for single instance evaluation in the encrypted domain.

In Fig. 4, we further break down the processing time for the average instance into three steps: encryption of the input data, inference (evaluation of the neural network), and decryption of the encrypted results. Our analysis revealed that encryption and inference typically account for a larger portion of the overall processing time, with a ratio of approximately 1 : 9. Although expected for inference to be more time-consuming for activation functions with higher multiplicative depths, this trend was also observed for the encryption step^51^. Decryption proved to be a relatively efficient operation, contributing to the overall computation time by less than 0.01 seconds.

## Conclusion

In this paper, we proposed a biometric recognition system that leverages homomorphic encryption for enhancing data privacy. The system’s architecture consists of a feature extractor and a classifier. The FE runs locally and only sends sensitive data to the computation node after it has been encrypted. The classifier operates on encrypted inputs and returns the result which can only be decrypted with the data owner’s private key.

As a practical demonstration, we applied our HE-based biometric authentication system to the CASIA-B gait dataset. We employed a self-supervised vision transformer model as the FE and trained three simple FCNN classifiers on plaintext data, each with a different activation function ($f_\text {Linear}$, $f_\text {ReLU}$, and $f_\text {Sigmoid}$). In our experiments, we compared the performance of the classifiers in both plaintext and encrypted domains. In the case of encrypted evaluation, the classifiers needed to be redesigned to be HE-friendly, which primarily referred to replacing activation functions with their polynomial approximations.

We initially investigated the performance of the three core activation functions and their polynomial approximations in the plaintext domain. The sigmoid activation function $f_\text {Sigmoid}$ yielded the best results for this task, followed by $f_\text {ReLU}$, and then $f_\text {Linear}$. Polynomial approximation further improved the performance for $\text {CLS}_\text {Sigmoid'}$, but it had a negative effect on the performance of $\text {CLS}_\text {ReLU'}$. Then, we assessed the impact of HE on classification accuracy and computational overhead. We noted a slight decrease in accuracy for evaluations conducted in the encrypted domain and observed a linear correlation between the multiplicative depth of the activation function and the processing time, which averaged 8–12 s for per-instance evaluation. Although these times could become a bottleneck in real-time or high-throughput scenarios, the current implementation of the TenSEAL framework could benefit from advances in GPU parallelization for HE and achieve significant performance improvements in the near future.

We have shown that it is possible to protect sensitive biometric data while maintaining accurate classification performance using HE. Future work could explore the broader applicability of our findings to other biometric modalities and datasets, as well as investigate the impact of different parameters of the HE scheme, particularly through empirical evaluation. Additionally, examining other HE schemes that enable the use of arbitrary activation functions without approximations like TFHE^13^ could further enhance the performance and practicality of our approach.

## Data Availability

The dataset used in this study is owned by the Center for Biometrics and Security Research, Institute of Automation, Chinese Academy of Sciences, and can be requested from the original authors. Other materials related to this study are available from the corresponding author upon request.

## References

[1] Munusamy, V. & Senthilkumar, S. Emerging trends in gait recognition based on deep learning: A survey. *PeerJ Comput. Sci.*10.7717/peerj-cs.2158 (2024). 39145199 10.7717/peerj-cs.2158 PMC11323174

[2] Aung, S. T. Y. & Kusakunniran, W. A comprehensive review of gait analysis using deep learning approaches in criminal investigation. *PeerJ Comput. Sci.*10.7717/peerj-cs.2456 (2024). 39650492 10.7717/peerj-cs.2456 PMC11622936

[3] Shen, C., Yu, S., Wang, J., Huang, G. Q. & Wang, L. A comprehensive survey on deep gait recognition: Algorithms, datasets, and challenges. *IEEE Trans. Biometr. Behav. Identity Sci.*10.1109/TBIOM.2024.3486345 (2025).

[4] Meden, B. et al. Privacy-enhancing face biometrics: A comprehensive survey. *IEEE Trans. Inf. Forensics Secur.*10.1109/TIFS.2021.3096024 (2021).

[5] Rivest, R. L., Shamir, A. & Adleman, L. A method for obtaining digital signatures and public-key cryptosystems. *Commun. ACM*10.1145/359340.359342 (1978).

[6] Gentry, C. Fully homomorphic encryption using ideal lattices. In *41st Annual ACM Symposium on Theory of Computing* 169–178. 10.1145/1536414.1536440 (2009).

[7] Cheon, J. H., Kim, A., Kim, M. & Song, Y. Homomorphic encryption for arithmetic of approximate numbers. In *International Conference on the Theory and Application of Cryptology and Information Security* 409–437. 10.1007/978-3-319-70694-8_15 (2017).

[8] Benaissa, A., Retiat, B., Cebere, B. & Belfedhal, A. E. TenSEAL: A library for encrypted tensor operations using homomorphic encryption. 10.48550/arXiv.2104.03152 (2021).

[9] Pinčić, D., Sušanj, D. & Lenac, K. Gait recognition with self-supervised learning of gait features based on vision transformers. *Sensors*10.3390/s22197140 (2022). 36236238 10.3390/s22197140 PMC9571216

[10] Schlögl, A. & Böhme, R. eNNclave: Offline inference with model confidentiality. In *Proceedings of the 13th ACM Workshop on Artificial Intelligence and Security* 93–104. 10.1145/3411508.3421376 (2020).

[11] Nocker, M., Drexel, D., Rader, M., Montuoro, A. & Schöttle, P. HE-MAN—Homomorphically Encrypted MAchine learning with oNnx models. In *Proceedings of the 2023 8th International Conference on Machine Learning Technologies* 35–45. 10.1145/3589883.3589889 (2023).

[12] Nikfam, F., Casaburi, R., Marchisio, A., Martina, M. & Shafique, M. A homomorphic encryption framework for privacy-preserving spiking neural networks. *Information*10.3390/info14100537 (2023).

[13] Stoian, A. et al. Deep neural networks for encrypted inference with TFHE. In *Cyber Security, Cryptology, and Machine Learning* 493–500. 10.1007/978-3-031-34671-2_34 (2023).

[14] Marcolla, C. et al. Survey on fully homomorphic encryption, theory, and applications. *Proc. IEEE*10.1109/JPROC.2022.3205665 (2022).

[15] Ameur, Y., Bouzefrane, S. & Audigier, V. *Application of Homomorphic Encryption in Machine Learning* 391–410. 10.1007/978-3-031-09640-2_18 (Springer, 2023).

[16] Blatt, M., Gusev, A., Polyakov, Y. & Goldwasser, S. Secure large-scale genome-wide association studies using homomorphic encryption. *Proc. Natl. Acad. Sci. U.S.A.*10.1073/pnas.1918257117 (2020). 32398369 10.1073/pnas.1918257117 PMC7261120

[17] Wood, A., Najarian, K. & Kahrobaei, D. Homomorphic encryption for machine learning in medicine and bioinformatics. *ACM Comput. Surv.*10.1145/3394658 (2020).

[18] Hong, S., Park, J. H., Cho, W., Choe, H. & Cheon, J. H. Secure tumor classification by shallow neural network using homomorphic encryption. *BMC Genom.*10.1186/s12864-022-08469-w (2022). 10.1186/s12864-022-08469-w PMC8994372 35395714

[19] Onoufriou, G., Hanheide, M. & Leontidis, G. EDLaaS: Fully homomorphic encryption over neural network graphs for vision and private strawberry yield forecasting. *Sensors*10.3390/s22218124 (2022). 36365823 10.3390/s22218124 PMC9658784

[20] Jia, H. et al. Efficient and privacy-preserving image classification using homomorphic encryption and chunk-based convolutional neural network. *J. Cloud Comput.*10.1186/s13677-023-00537-0 (2023).

[21] Yang, W., Wang, S., Cui, H., Tang, Z. & Li, Y. A review of homomorphic encryption for privacy-preserving biometrics. *Sensors*10.3390/s23073566 (2023). 37050626 10.3390/s23073566 PMC10098691

[22] Drozdowski, P., Buchmann, N., Rathgeb, C., Margraf, M. & Busch, C. On the application of homomorphic encryption to face identification. In *2019 International Conference of the Biometrics Special Interest Group (BIOSIG)* 1–5 (2019)

[23] Pradel, G. & Mitchell, C. Privacy-preserving biometric matching using homomorphic encryption. In *2021 IEEE 20th International Conference on Trust, Security and Privacy in Computing and Communications (TrustCom)* 494–505. 10.1109/TrustCom53373.2021.00079 (2021).

[24] Yang, Y. et al. Design on face recognition system with privacy preservation based on homomorphic encryption. *Wireless Pers. Commun.*10.1007/s11277-021-09311-4 (2022).

[25] Engelsma, J. J., Jain, A. K. & Boddeti, V. N. HERS: Homomorphically encrypted representation search. *IEEE Trans. Biometr. Behav. Identity Sci.*10.1109/TBIOM.2021.3139866 (2022).

[26] Morampudi, M. K., Prasad, M. V. N. K. & Raju, U. S. N. Privacy-preserving iris authentication using fully homomorphic encryption. *Multimedia Tools Appl.*10.1007/s11042-020-08680-5 (2020).

[27] Kumar, M. M., Prasad, M. & Raju, U. S. N. BMIAE: Blockchain-based multi-instance iris authentication using additive ElGamal homomorphic encryption. *IET Biometr.*10.1049/iet-bmt.2019.0169 (2020).

[28] Gomez-Barrero, M., Maiorana, E., Galbally, J., Campisi, P. & Fierrez, J. Multi-biometric template protection based on homomorphic encryption. *Pattern Recogn.*10.1016/j.patcog.2017.01.024 (2017).

[29] Sperling, L., Ratha, N., Ross, A. & Boddeti, V. N. HEFT: Homomorphically encrypted fusion of biometric templates. In *2022 IEEE International Joint Conference on Biometrics (IJCB)* 1–10. 10.1109/IJCB54206.2022.10007995 (2022).

[30] Singh, S., Igene, L. & Schuckers, S. Securing biometric data: Fully homomorphic encryption in multimodal iris and face recognition. 10.48550/arXiv.2408.14609 (2024).

[31] Vallabhadas, D. K., Sandhya, M., Sarkar, S. & Chandra, Y. R. Multimodal biometric authentication using fully homomorphic encryption. In *2023 2nd International Conference on Paradigm Shifts in Communications Embedded Systems, Machine Learning and Signal Processing (PCEMS)* 1–6. 10.1109/PCEMS58491.2023.10136104 (2023).

[32] Lin, L., Tian, B., Zhao, Y. & Niu, Y. A privacy-preserving gait recognition scheme under homomorphic encryption. In *2022 International Conference on Networking and Network Applications (NaNA)* 406–410. 10.1109/NaNA56854.2022.00075 (2022).

[33] Castro, F., Impedovo, D. & Pirlo, G. A hybrid protection scheme for the gait analysis in early dementia recognition. *Sensors*10.3390/s24010024 (2024). 38202886 10.3390/s24010024 PMC10780691

[34] Naz, A. et al. Privacy-preserving abnormal gait detection using computer vision and machine learning. *EAI Endors. Trans. Pervas. Health Technol.*10.4108/eetpht.11.9094 (2025).

[35] Delgado-Santos, P. et al. GaitPrivacyON: Privacy-preserving mobile gait biometrics using unsupervised learning. *Pattern Recogn. Lett.*10.1016/j.patrec.2022.07.015 (2022).

[36] Parashar, A. & Shekhawat, R. S. Protection of gait data set for preserving its privacy in deep learning pipeline. *IET Biometr.*10.1049/bme2.12093 (2022).

[37] Su, Y., Li, Y. & Cao, Z. Gait-based privacy protection for smart wearable devices. *IEEE Internet Things J.*10.1109/JIOT.2023.3296650 (2024).

[38] Arman, S. M. et al. A comprehensive survey for privacy-preserving biometrics: Recent approaches, challenges, and future directions. *Comput. Mater. Continua*10.32604/cmc.2024.047870 (2024).

[39] Xu, W. et al. PrivGait: An energy-harvesting-based privacy-preserving user-identification system by gait analysis. *IEEE Internet Things J.*10.1109/JIOT.2021.3089618 (2022).36059439

[40] Van hamme, T., Rúa, E. A., Preuveneers, D. & Joosen, W. On the security of biometrics and fuzzy commitment cryptosystems: A study on gait authentication. *IEEE Trans. Inf. Forensics Secur.*10.1109/TIFS.2021.3124735 (2021).

[41] McConvey, J. R. Worldcoin open-sources multi-party computation system. BiometricUpdate.com. https://www.biometricupdate.com/202405/worldcoin-open-sources-multi-party-computation-system (Accessed 14 June) (2025)

[42] Geng, Y., Yuan, M., Wen, X., Wang, Y. & Meng, L. Distributed learning for gait-based human recognition. In *2024 International Conference on Identification, Information and Knowledge in the Internet of Things (IIKI)* 37–42. 10.1109/IIKI65561.2024.00016 (2024)

[43] Zakariyya, I., Tran, L., Sivangi, K. B., Henderson, P. & Deligianni, F. Differentially private integrated decision gradients (IDG-DP) for radar-based human activity recognition. In *2025 IEEE/CVF Winter Conference on Applications of Computer Vision (WACV)* 5611–5622. 10.1109/WACV61041.2025.00548 (2025).

[44] Yu, S., Tan, D. & Tan, T. A framework for evaluating the effect of view angle, clothing and carrying condition on gait recognition. In *18th International Conference on Pattern Recognition (ICPR’06)* 441–444. 10.1109/ICPR.2006.67 (2006).

[45] Han, J. & Bhanu, B. Individual recognition using gait energy image. *IEEE Trans. Pattern Anal. Mach. Intell.*10.1109/TPAMI.2006.38 (2006). 16468626 10.1109/TPAMI.2006.38

[46] Caron, M. et al. Emerging properties in self-supervised vision transformers. In *Proceedings of the IEEE/CVF International Conference on Computer Vision* 9630–9640. 10.1109/ICCV48922.2021.00951 (2021).

[47] Ali, R. E., So, J. & Avestimehr, A. S. On Polynomial approximations for privacy-preserving and verifiable ReLU networks. 10.48550/arXiv.2011.05530 (2024).

[48] Chen, H. et al. Logistic regression over encrypted data from fully homomorphic encryption. *BMC Med. Genom.*10.1186/s12920-018-0397-z (2018). 10.1186/s12920-018-0397-z PMC6180402 30309350

[49] Lee, J.-W. et al. Privacy-preserving machine learning with fully homomorphic encryption for deep neural network. *IEEE Access*10.1109/ACCESS.2022.3159694 (2022).

[50] Meftah, S. et al. DOReN: Toward efficient deep convolutional neural networks with fully homomorphic encryption. *IEEE Trans. Inf. Forensics Secur.*10.1109/TIFS.2021.3090959 (2021).

[51] Lee, J., Duong, P. N. & Lee, H. Configurable encryption and decryption architectures for CKKS-based homomorphic encryption. *Sensors*10.3390/s23177389 (2023). 37687844 10.3390/s23177389 PMC10490559

